# Does Migration Limit the Effect of Health Insurance on Hypertension Management in China?

**DOI:** 10.3390/ijerph14101256

**Published:** 2017-10-20

**Authors:** Hai Fang, Yinzi Jin, Miaomiao Zhao, Huyang Zhang, John A. Rizzo, Donglan Zhang, Zhiyuan Hou

**Affiliations:** 1 China Center for Health Development Studies, Peking University, Beijing 100000, China; hfang@hsc.pku.edu.cn (H.F.); lhjinyinzi@163.com (Y.J.); huyangzhang@bjmu.edu.cn (H.Z.); 2 Department of Health Policy and Administration, School of Public Health, Peking University, Beijing 100191, China; miaomiao.zhao@bjmu.edu.cn; 3 Department of Economics, State University of New York at Stony Brook, Stony Brook, NY 11794, USA; John.Rizzo@stonybrookmedicine.edu; 4 Department of Preventive Medicine, State University of New York at Stony Brook, Stony Brook, NY 11794, USA; 5 Department of Health Policy and Management, College of Public Health, University of Georgia, Athens, GA 30602, USA; dzhang@uga.edu; 6 Department of Social Medicine, School of Public Health, National Key Laboratory of Health Technology Assessment (National Health and Family Planning Commission), Collaborative Innovation Center of Social Risks Governance in Health, Fudan University, 138 Yi Xue Yuan Road, Shanghai 200032, China

**Keywords:** migration, health insurance, hypertension, awareness, disease control, China

## Abstract

*Background:* In China, rapid urbanization has caused migration from rural to urban areas, and raised the prevalence of hypertension. However, public health insurance is not portable from one place to another, and migration may limit the effectiveness of this non-portable health insurance on healthcare. Our study aims to investigate whether migration limits the effectiveness of health insurance on hypertension management in China. *Methods:* Data were obtained from the national baseline survey of the China Health and Retirement Longitudinal Study in 2011, including 4926 hypertensive respondents with public health insurance. Outcome measures included use of primary care, hypertension awareness, medication use, blood pressure monitoring, physician advice, and blood pressure control. Multivariate logistic regressions were estimated to examine whether the effects of rural health insurance on hypertension management differed between those who migrated to urban areas and those who did not migrate and lived in rural areas. *Results:* Among hypertensive respondents, 60.7% were aware of their hypertensive status. Compared to rural residents, the non-portable feature of rural health insurance significantly reduced rural-to-urban migrants’ probabilities of using primary care by 7.8 percentage points, hypertension awareness by 8.8 percentage points, and receiving physician advice by 18.3 percentage points. *Conclusions:* In China, migration to urban areas limited the effectiveness of rural health insurance on hypertension management due to its non-portable nature. It is critical to improve the portability of rural health insurance, and to extend urban health insurance and primary care coverage to rural-to-urban migrants to achieve better chronic disease management.

## 1. Introduction

Since the mid-1980s, China has experienced the most rapid urbanization and internal migration in human history [1]. Migrants had reached approximately 247 million in 2015, accounting for 18% of total Chinese population [2]. Such rapid urbanization undoubtedly had important consequences for population health [1,3]. While offering higher income, evidence from the 1991–2004 China Health and Nutrition Survey indicated that urbanization had negative effects on health and raised the prevalence of chronic diseases (such as hypertension) due to increased exposure to risk factors [4]. There were larger increases in blood pressure among rural-to-urban migrants than their rural counterparts [5].

In China, the prevalence of hypertension increased dramatically from 18% in 2002 to 34% in 2010 [6,7], and this prevalence was higher among rural-to-urban migrants, approximately 80 million of whom had hypertension in 2012 [5]. It has been estimated that, nationally, 450,000 deaths were attributable to hypertension in 2010, and direct healthcare costs due to hypertension totaled 9.7 billion U.S. Dollars, accounting for 3.4% of total healthcare expenditure [8,9]. However, hypertension management remained poor in China [10,11], although it can be controlled at fairly low cost [12]. In 2010, just 36% of hypertensive adults were aware of their hypertension status and only 17% controlled their hypertension well [7]. This may be explained by both residents’ health awareness and the incentive system for healthcare providers. On the one hand, most Chinese residents have poor levels of education, they believe they are healthy and see no value in health checks. They also have a free choice when seeking healthcare, and always bypass the community healthcare centers and visit hospitals directly. This means that free health checks are not used, although they should be offered by community healthcare centers. Therefore, many Chinese are unaware of their health and hypertension status. On the other hand, under the fee-for-services payment system, physicians have strong incentives to provide medical care rather than preventive care, and so they are unwilling to offer preventive care, including hypertension management services.

However, the urban health systems have not focused on the management of chronic diseases such as hypertension for migrants as an important public health challenge [13,14]. Although rural-to-urban migrants live in urban areas, they still retain their official rural residency status due to the permanent residence registration system (“Hukou”). Therefore, they cannot be entitled to the same social benefits that permanent urban residents enjoy, including access to health insurance and healthcare services in urban areas [15]. The Hukou system classifies people as rural or urban residents based on their original locations and it is not easy to transfer from rural to urban official residency [16]. Currently, China’s public health insurance programs are also designed according to the Hukou system and/or employment status, and are not portable from one place to another [17,18]. The rural health insurance program is targeted to people with a rural Hukou, while the urban health insurance programs are designed for permanent non-employee urban residents or those with formal employment in urban areas respectively. The rural-to-urban migrants without formal employment are generally ineligible for urban health insurance, but must rely on their rural health insurance. Although there is better access to care, and higher-quality care in urban areas than rural areas in general, migrants face obstacles to use urban healthcare. If they seek healthcare in urban health facilities, it is difficult to get reimbursement from their rural health insurance program since urban facilities are out of their insurance coverage. Therefore, migration may limit the effect of health insurance on healthcare access for rural-to-urban migrants [13,17,19].

Because migration may raise the hypertension prevalence, but limit the effect of the non-portable health insurance, it is important to investigate the role of migration on the effect of health insurance on hypertension management in China. While some studies have examined associations of migration and health status [1,4,19], there is little evidence on healthcare utilization among rural-to-urban migrants [15,17], and no research has been focused on hypertension management of migrants in China. Using a nationally representative survey from 2011, the present study aimed to bridge this gap by evaluating the effects of migration and health insurance on hypertension management, and their interaction effects. The interaction effects of migration and health insurance were used to examine whether the effectiveness of rural health insurance on hypertension management differed between those who migrated to urban areas and those who did not migrate and lived in rural areas. Our findings have potentially important implications for improved chronic disease management and improving the health of migrants in China.

## 2. Methods

### 2.1. Study Design and Data

We used data from the China Health and Retirement Longitudinal Study (CHARLS) [20], which is publicly available (The CHARLS data can be downloaded from the following website: http://charls.pku.edu.cn/en). The national baseline survey of CHARLS was conducted through a four-stage probability sampling technique between June 2011 and March 2012. In the first sampling stage, 150 county-level units were randomly chosen with the probability proportional to size (PPS) from a sampling frame containing all county-level units of China excluding those in Tibet. In the second stage, three communities were randomly chosen by the PPS method within each county-level unit. For each community, 24 households with members aged 45 or older were randomly selected. The member aged 45 or older and his or her spouse (if present) were interviewed in each household, resulting in a total sample of 17,708 respondents.

Data were collected through interviews and physical measurements. The CHARLS questionnaire covered social demographics, health insurance, health status, health behaviors, and hypertension management. Physical measurements included blood pressure (BP), height, and weight. Among all respondents, 13,708 respondents provided complete BP measures. In our analysis, we only used hypertensive respondents with public health insurance, leaving a study sample of 4926 individuals.

### 2.2. Measures of Hypertension Management

For each respondent, BP was measured three times (at least 45 s apart) in the sitting position after a 5-min rest. The mean of the three readings was then calculated as their BP value. A person is considered to be hypertensive if the mean systolic BP is greater than or equal to 140 mmHg and/or the mean diastolic BP is greater than or equal to 90 mmHg. Consistent with the medical literature, respondents with a previous diagnosis of hypertension by a doctor and/or self-reported current treatment with anti-hypertensive medication are also regarded as hypertensive [21,22].

The relevant outcome measures in the present study included use of primary care and hypertension management. Although it may not be a direct measure of hypertension management, access to primary care helps promote patients’ understanding and awareness of their hypertension status. According to the Chinese Guidelines for the Prevention and Treatment of Hypertension [22], hypertensive respondents were defined as being aware of their hypertension if they were previously diagnosed as hypertensive by physicians or self-reported to be hypertensive. For those respondents who were aware of their hypertension, the survey included the following indicators of hypertension management: medication use, BP monitoring, physician advice, and BP control. Detailed definitions of these measures are provided in Table 1.

### 2.3. Statistical Analysis

Descriptive analyses presented the proportion of respondents utilizing hypertension management. Rural-to-urban migrants were defined as respondents with rural health insurance but currently living in urban areas. Generally, they migrated to and lived in the urban areas for more than 6 months, but they were not eligible for urban Hukou (permanent residents registration system) and cannot be seen as officially permanent urban residents. To identify the effect of migration and health insurance on hypertension management, multivariate logistic regressions were estimated to control for potential confounding factors, including gender, age, education, marital status, household income, household size, self-assessed health and other chronic diseases [23]. Fixed effects were also used in all regressions to adjust for unobserved provincial-level factors. We also estimated the results separately for males and females.

Two models for each outcome variable, with and without interaction terms [24] between the type of health insurance and place of residence (rural or urban), were estimated separately. The interaction term was used to examine whether the association of the type of health insurance with hypertension management differed by place of residence, thus helping to capture the effects of migration. Odds ratios (ORs) with 95% confidence intervals (CIs) were reported. We also computed the mean marginal effects of the interaction term between migration and rural health insurance for logistic models using the *inteff* (in STATA) command [25,26]. All statistical analyses were performed using STATA 12.0 (StataCorp, College Station, TX, USA).

## 3. Results

### 3.1. Characteristics of the Study Sample

Table 1 reports descriptive statistics on the hypertension management measures. Among all the hypertensive respondents, 21.1% had primary care in the previous month, and 60.7% were aware of their hypertensive status. Of those aware of their hypertension, 76.7% were receiving anti-hypertensive medications, 57.9% monitored blood pressure at least once every three months, 54.9% had received physician advice about their hypertensive treatments, but only 42.3% had their BP controlled in the normal range.

Table 2 presents the characteristics of the hypertensive respondents comprising our study sample. Among a total of 4926 hypertensive respondents, 82.5% had rural health insurance, but only 62.0% were living in rural areas. This indicated that about 20% respondents with rural health insurance had migrated to urban areas, defined as rural-to-urban migrants. Females comprised 55.2% of the sample and the average age was 62 years old.

### 3.2. Effects of Migration and Health Insurance on Hypertension Management

Table 3 shows the results of multivariate logistic regressions estimating the effects of rural-to-urban migration and health insurance on each outcome of hypertension management. There are two models for each outcome. Compared to Model 1, we added the interaction between urban residential areas and rural health insurance in Model 2 to test whether the effects of rural health insurance on hypertension management differed between those who migrated to urban areas and those who did not migrate and lived in rural areas. According to the results in Table 3, the ORs of the interaction term are less than one for each outcome, indicating poorer hypertension management for rural-to-urban migrants (although some differences in outcomes were statistically insignificant).

As shown in Model 1 in Table 3, neither residing in urban areas nor having rural health insurance were significantly associated with primary care utilization. After adding the interaction term in Model 2, the holders of rural health insurance living in rural areas became much more likely to use primary care (OR = 1.96, 95% CI: 1.07–3.60, *p* = 0.029), and the OR for the interaction term between urban residency and rural health insurance was 0.54 (95% CI = 0.28–1.05, *p* = 0.070), indicating that the effect of rural health insurance for migrants to urban areas was 46% smaller than its effect on rural respondents remaining in the rural areas. A similar effect of migration was also observed for hypertension awareness. The OR for the interaction term on awareness was 0.65 (95% CI = 0.40–1.07, *p* = 0.070), indicating that migrating to urban areas reduces the probability of being aware of their hypertension status.

With respect to medication use, BP monitoring and BP control, Model 2 in Table 3 shows no significant differences by place of residence and health insurance plans, and the effects of rural health insurance did not vary significantly by migration status. However, hypertensive residents living in urban areas were much more likely to receive physician advice than those living in rural areas (OR = 2.75, 95% CI: 1.55–4.89, *p* = 0.001), and rural health insurance for those living in rural areas enhances the likelihood of obtaining physician advice (OR = 1.61, 95% CI: 0.93–2.79, *p* = 0.090). The non-portable nature of rural health insurance limits the chance of obtaining physician advice for rural-to-urban migrants (OR = 0.45, 95% CI: 0.24–0.83, *p* = 0.010).

To more intuitively understand the interaction effects, we also calculated the mean marginal effects of the interaction term between migration and rural health insurance from Table 3, and the results of marginal effects are presented in Table 4. The effects of rural health insurance on hypertension management were of limited benefit to rural-to-urban migrants, especially for primary care, awareness and physician advice. The marginal effects of the interaction term showed that compared to rural residents, the non-portable feature of rural health insurance reduced rural-to-urban migrants’ probabilities of receiving primary care by 7.8 percentage points, hypertension awareness by 8.8 percentage points, and receiving physician advice by 18.3 percentage points, after controlling for other variables.

In addition, we estimated the results separately for males/females. We find that the interaction effects of living in an urban area and rural health insurance differed by gender. The interaction effects were insignificant among hypertensive males, whereas these interaction effects on primary care, awareness and BP control were significant among hypertensive females, negatively affecting their hypertension management due to rural-to-urban migration (Table 5).

## 4. Discussion

To our knowledge, this is the first study to evaluate the association between migration, health insurance and hypertension management in China. Using the 2011 CHARLS national survey, we found that up to 40% were unaware of their hypertension status. Conditional to respondents’ hypertension awareness, nearly 80% received anti-hypertensive medications and more than half had obtained physician advice on healthy behaviors, resulting in 42% achieving effective blood pressure control. After adjusting for confounding factors, migration limited the effect of health insurance on hypertension management due to the non-portable nature of rural health insurance, which mainly occurred in terms of reduced primary care, awareness and physician advice.

We found that rural-to-urban migration limited the benefits of rural health insurance, although public health insurance can promote better management of hypertension [27]. In particular, the benefits of rural health insurance on primary care utilization were significantly smaller for migrants compared to local rural residents, resulting in lower awareness and worse management of their hypertension. The main reason for these results is that in China, rural and urban health insurance programs are highly segmented and cannot be transferred across geographic areas [28,29]. Rural-to-urban migrants only qualify for rural health insurance but not urban health insurance, which is only offered to permanent urban residents with urban Hukou or those with formal employment residing in urban areas. In general, rural health insurance mainly covers healthcare in designated facilities within rural hometowns, with little reimbursement for health services in the urban areas (out of network) where migrants live [17]. Because these migrants live in urban areas, it imposes additional obstacles for migrants to return to their rural hometown to obtain health services. If they use healthcare in urban areas, they cannot obtain full reimbursement from either urban or rural health insurance. This reduces migrants’ access to both urban and rural healthcare, resulting in poorer management of their hypertension. In order to improve hypertension management for these migrants, it is critical to improve the portability of rural health insurance and extend urban health insurance to cover rural-to-urban migrants in the short term, and further integrate rural and urban health insurance programs as a long-term solution [28]. Fortunately, some provinces and cities have started to integrate these different health insurance programs in local areas, such as Chengdu and Chongqing Cities [30]. Evidence suggests that this integrated system may improve healthcare benefits for vulnerable populations [31]. 

Specifically, the non-portable feature of rural health insurance mainly reduces rural-to-urban migrants’ probabilities of using primary care, being aware of their condition, and receiving physician advice, but has no significant influence on other dimensions of hypertension management. A plausible explanation is that primary care, awareness and physician advice relies more on access to primary healthcare services and healthcare professionals, but taking medication and monitoring blood pressure are, to a greater extent, based on self-management. These findings are consistent with previous studies showing that migrants underutilize health services both in their hometown and destination cities [15,32]. Unfortunately, migrant health and chronic disease management have received far less attention in the urban healthcare system [13]. Our study has documented the poor chronic disease management among migrants for hypertension due to the non-portable feature of rural health insurance coverage, a pattern that may well be repeated for many other chronic diseases. To alleviate these disparities, it is necessary to offer equal benefits to migrants and locals, and to deliver urban primary healthcare to migrants [33]. This will improve migrants’ healthcare access and allow healthcare professionals to play more active roles in migrant health, especially in the management of chronic diseases.

In addition, a World Health Organization study found that among similar middle-income countries, hypertension management in China was poorer than Russia, India and Mexico, but better than South Africa between 2007 and 2010 [34]. Equally, it was much poorer than the developed countries, especially in terms of hypertension awareness [27]. Our study revealed that up to 40% of subjects were unaware of their hypertension in 2011, which implied that nearly 40 million people aged 45 and above in China had hypertension but remained undiagnosed. Since the costs incurred by hypertension management are low, China should prevent and control hypertension as well as other developing or more developed countries. For example, in North America, Europe and Japan, awareness and treatment rates attained 80%, and approximately 60% can achieve effective blood pressure control [35,36]. Hypertension awareness, which is a pre-condition for the prevention and control of hypertension, largely depends on the capacity of the healthcare system to diagnose chronic diseases [37]. Therefore, further health system improvements should focus on early detection of chronic diseases, especially for migrants [27]. 

We further found that the effects of migration on hypertension management are markedly worse for females than for males. Migrant females in China earn much less than migrant males and may lack sufficient resources to travel back to distant rural areas for their healthcare. Therefore, the separate urban—rural health insurance system created larger barriers for female migrants than males.

This study has several limitations. First, rural-to-urban migrants may differ from residents who remain in rural areas in ways that we were unable to observe and control for. For example, differences in disease severity and lifestyles (more stress for migrants) could explain some of our findings, but some of these important confounding factors were unobservable. To reduce these confounding effects, self-reported health status and the presence of other chronic diseases were included in models. Second, sample selection bias could be caused by missing values in the biomarker data. Fortunately, the characteristics of those with and without BP measures were not significantly different in the CHARLS dataset. Third, our sample covered the Chinese population aged 45 or above, and was not fully representative of China’s hypertensive population, since the hypertension prevalence among China’s 18–44 age group was at a substantial level of 17.5% in 2010 [38]. Finally, we only estimated the effects on hypertension management, but did not look at other chronic diseases for a robust check. In the future, we will extend our study to other chronic diseases, and conduct qualitative studies to understand why migrants underutilize treatment services.

## 5. Conclusions

The benefits of rural health insurance to hypertension management were significantly smaller for rural-to-urban migrants compared to local rural residents. Although migration may enhance their socioeconomic status, rural-to-urban migration limited the benefits of rural health insurance due to its non-portable nature. Many rural-to-urban migrants were unaware of their hypertension, because they were unable to use the outpatient services in the urban areas due to the separate health insurance network [39]. It is critical to improve the portability of rural health insurance and to extend urban health insurance and primary care coverage to rural-to-urban migrants to achieve better chronic disease management, especially early detection of chronic diseases to improve their awareness.

## Figures and Tables

**Table 1:** Definition and description of hypertension management.

Outcome Measures	Definitions	Percent (%)
**All hypertensive respondents (*N* = 4926)**		
Primary care	1, if the respondent has visited healthcare institutions or been visited by physicians in the last month; 0, otherwise	21.1
Awareness	1, if the respondent was previously diagnosed as hypertension by physicians or self-reported to be hypertensive; 0, otherwise	60.7
**Hypertensive aware respondents (*N* = 2976)**		
Medication use	1, if the respondent was receiving anti-hypertensive medications; 0, otherwise	76.7
BP monitor	1, if the respondent has monitored BP at least once every three months during last year; 0, otherwise	57.9
Physician advice	1, if the respondent has ever gotten health advice from physicians on weight control, exercise, diet and/or smoking cessation; 0, otherwise	54.9
BP control	1, if the respondent’s systolic BP is less than 140 mmHg and diastolic BP is less than 90 mmHg; 0, otherwise	42.3

Note: BP indicates blood pressure.

**Table 2:** Characteristics of the hypertensive respondents (*N* = 4926).

Characteristics	Percent (%)
Public health insurance	
Rural health insurance	82.5
Urban health insurance	17.5
Place of residence	
Rural living	62.0
Urban living	38.0
Region	
East	35.6
Central	33.0
West	31.4
Household income per capita (mean, SD)	9328.5 (13,921.3)
Household size (mean, SD)	3.5 (1.8)
Gender	
Male	44.8
Female	55.2
Age, years	
45–54	23.9
55–64	38.7
65–74	25.1
75+	12.2
Education	
No education	33.0
No education but can read/write	18.5
Primary school	22.3
Junior high school and above	26.1
Marital status	
Currently Married	83.2
Divorced and others	16.8
Self-assessed health	
Good	18.1
Fair	46.1
Poor	35.9
Having other chronic diseases	
Yes	67.2
No	32.8

**Table 3:** Adjusted odds ratios (95% Confidence Interval (CI)) for hypertension management by logistic regression.

Variables	Primary Care		Awareness		Medication Use		BP Monitor		Physician Advice		BP Control	
	Model 1	Model 2	Model 1	Model 2	Model 1	Model 2	Model 1	Model 2	Model 1	Model 2	Model 1	Model 2
Urban living	0.97	1.69	1.03	1.51 *	1.48 ***	1.55	1.37 ***	1.40	1.35 ***	2.75 ***	0.83 *	0.92
	(0.81–1.16)	(0.90–3.19)	(0.88–1.20)	(0.94–2.42)	(1.18–1.85)	(0.80–3.01)	(1.13–1.68)	(0.76–2.59)	(1.11–1.64)	(1.55–4.89)	(0.68–1.00)	(0.52–1.63)
Rural health insurance	1.20	1.96 **	0.71 ***	0.99	0.78	0.82	0.70 ***	0.72	0.84	1.61 *	0.82	0.91
	(0.93–1.53)	(1.07–3.60)	(0.57–0.87)	(0.64–1.55)	(0.58–1.06)	(0.44–1.53)	(0.54–0.92)	(0.40–1.29)	(0.66–1.08)	(0.93–2.79)	(0.65–1.05)	(0.53–1.57)
Rural health insurance * Urban living		0.54 *		0.65 *		0.95		0.98		0.45 **		0.88
		(0.28–1.05)		(0.40–1.07)		(0.47–1.91)		(0.51–1.87)		(0.24–0.83)		(0.48–1.61)
Log income	1.01	1.01	1.01	1.01	1.01	1.01	1.02	1.02	1.01	1.01	1.00	1.00
	(0.98–1.03)	(0.98–1.03)	(0.99–1.04)	(0.99–1.04)	(0.98–1.05)	(0.98–1.05)	(0.99–1.05)	(0.99–1.05)	(0.98–1.04)	(0.98–1.04)	(0.97–1.03)	(0.97–1.03)
Household size	1.01	1.01	0.97	0.97	0.98	0.98	1.01	1.01	1.00	1.00	1.00	1.00
	(0.97–1.05)	(0.97–1.06)	(0.94–1.01)	(0.94–1.01)	(0.93–1.04)	(0.93–1.04)	(0.96–1.06)	(0.96–1.06)	(0.95–1.04)	(0.95–1.04)	(0.95–1.05)	(0.95–1.05)
Female	1.24 ***	1.23 **	1.29 ***	1.29 ***	1.30 ***	1.30 ***	1.22 **	1.21 **	0.56 ***	0.55 ***	1.05	1.05
	(1.05–1.45)	(1.04–1.44)	(1.13–1.48)	(1.12–1.47)	(1.07–1.59)	(1.07–1.59)	(1.02–1.45)	(1.02–1.45)	(0.47–0.66)	(0.46–0.65)	(0.89–1.24)	(0.89–1.24)
Age (referance group: 45–54)												
55–64	0.98	0.98	1.31 ***	1.31 ***	1.66 ***	1.66 ***	1.55 ***	1.55 ***	0.97	0.97	0.93	0.93
	(0.81–1.20)	(0.81–1.20)	(1.11–1.54)	(1.11–1.54)	(1.32–2.10)	(1.32–2.10)	(1.26–1.92)	(1.26–1.92)	(0.79–1.19)	(0.79–1.19)	(0.76–1.13)	(0.76–1.13)
65–74	0.98	0.98	1.14	1.14	1.97 ***	1.97 ***	1.43 ***	1.43 ***	0.92	0.92	0.82 *	0.82*
	(0.78–1.23)	(0.78–1.23)	(0.95–1.38)	(0.95–1.38)	(1.50–2.60)	(1.50–2.60)	(1.12–1.83)	(1.12–1.83)	(0.72–1.16)	(0.73–1.17)	(0.65–1.04)	(0.65–1.04)
75+	1.07	1.07	1.05	1.05	3.02 ***	3.02 ***	1.39 **	1.39 **	0.90	0.90	0.43 ***	0.43 ***
	(0.80–1.44)	(0.80–1.44)	(0.82–1.34)	(0.82–1.34)	(2.03–4.49)	(2.03–4.49)	(1.00–1.93)	(1.00–1.94)	(0.65–1.24)	(0.65–1.23)	(0.31–0.60)	(0.31–0.60)
Education (referred to no education)												
No education but can read/write	1.00	1.01	1.27 **	1.28 **	1.09	1.09	1.10	1.10	0.96	0.97	1.13	1.13
	(0.81–1.25)	(0.81–1.25)	(1.06–1.53)	(1.06–1.54)	(0.83–1.43)	(0.83–1.43)	(0.87–1.39)	(0.87–1.39)	(0.76–1.21)	(0.77–1.22)	(0.90–1.42)	(0.90–1.43)
Primary school	1.05	1.05	1.24 **	1.24 **	1.19	1.19	1.44 ***	1.44 ***	1.08	1.09	1.08	1.08
	(0.84–1.31)	(0.84–1.32)	(1.03–1.49)	(1.03–1.49)	(0.90–1.57)	(0.90–1.57)	(1.13–1.83)	(1.13–1.83)	(0.86–1.37)	(0.86–1.37)	(0.86–1.36)	(0.86–1.36)
Junior high school and above	1.19	1.19	1.35 ***	1.34 ***	1.27	1.27	1.85 ***	1.85 ***	1.23 *	1.23	1.19	1.19
	(0.94–1.51)	(0.94–1.50)	(1.10–1.65)	(1.10–1.64)	(0.94–1.70)	(0.94–1.70)	(1.43–2.41)	(1.43–2.41)	(0.96–1.58)	(0.96–1.58)	(0.93–1.52)	(0.93–1.52)
Currently Married	0.92	0.92	1.24 **	1.24 **	0.97	0.97	0.87	0.87	1.08	1.08	1.06	1.06
	(0.75–1.13)	(0.75–1.14)	(1.04–1.47)	(1.04–1.48)	(0.74–1.29)	(0.74–1.29)	(0.68–1.10)	(0.68–1.10)	(0.86–1.35)	(0.87–1.36)	(0.84–1.33)	(0.84–1.33)
Self-assessed health (referred to good)												
Fair	1.75 ***	1.76 ***	1.60 ***	1.60 ***	1.34 **	1.34 **	1.06	1.06	1.06	1.06	1.06	1.06
	(1.35–2.28)	(1.35–2.28)	(1.35–1.89)	(1.35–1.89)	(1.03–1.75)	(1.03–1.75)	(0.83–1.36)	(0.83–1.36)	(0.83–1.34)	(0.84–1.34)	(0.84–1.34)	(0.84–1.34)
Poor	4.19 ***	4.19 ***	2.54 ***	2.54 ***	2.00 ***	2.00 ***	1.33 **	1.33 **	1.54 ***	1.54 ***	1.04	1.04
	(3.21–5.46)	(3.22–5.46)	(2.11–3.06)	(2.11–3.06)	(1.51–2.65)	(1.51–2.66)	(1.03–1.72)	(1.03–1.72)	(1.20–1.97)	(1.21–1.97)	(0.81–1.33)	(0.81–1.33)
Having other chronic diseases	1.69 ***	1.70 ***	1.92 ***	1.92 ***	1.17	1.17	1.54 ***	1.54 ***	1.37 ***	1.37 ***	0.99	0.99
	(1.42–2.03)	(1.42–2.03)	(1.68–2.19)	(1.68–2.19)	(0.95–1.44)	(0.95–1.44)	(1.28–1.86)	(1.28–1.86)	(1.15–1.64)	(1.14–1.64)	(0.82–1.18)	(0.82–1.18)
Constant	0.08 ***	0.05 ***	0.72	0.52 **	0.75	0.72	0.43 **	0.42 **	1.14	0.62	1.21	1.10
	(0.04–0.14)	(0.02–0.11)	(0.43–1.19)	(0.27–0.97)	(0.36–1.54)	(0.29–1.75)	(0.23–0.82)	(0.19–0.96)	(0.61–2.12)	(0.28–1.34)	(0.66–2.24)	(0.51–2.38)
Observations	4910	4910	4889	4889	2967	2967	2912	2912	2961	2961	2967	2967

Notes: All models include provincial fixed effects. BP denotes blood pressure. Significance level: *** *p* < 0.01, ** *p* < 0.05, * *p* < 0.10.

**Table 4:** Interaction effects between rural health insurance and urban living on hypertension management by logistic regression.

	Primary Care	Awareness	Medication Use	BP Monitor	Physician Advice	BP Control
Coefficient	−0.078	−0.088	−0.001	0.001	−0.183	−0.029
Standard error	0.040	0.054	0.057	0.070	0.071	0.073
*p*-value	0.052	0.091	0.985	0.980	0.010	0.696

Notes: All models include the same variables as Table 3. BP: denotes blood pressure.

**Table 5:** Adjusted odds ratios (95% CI) for hypertension management by logistic regression, male/female.

Variables	Primary Care		Awareness		Medication Use		BP Monitor		Physician Advice		BP Control	
	Male	Female	Male	Female	Male	Female	Male	Female	Male	Female	Male	Female
Urban living	1.11	5.55 **	1.44	1.96	1.65	1.10	1.12	2.59	2.19 **	3.45 *	0.66	7.24 *
	(0.53–2.30)	(1.17–26.29)	(0.82–2.55)	(0.76–5.03)	(0.74–3.66)	(0.26–4.72)	(0.54–2.34)	(0.69–9.69)	(1.11–4.29)	(0.94–12.66)	(0.34–1.28)	(0.88–59.36)
Rural health insurance	1.49	6.02 **	0.85	1.45	0.85	0.59	0.69	1.12	1.48	1.76	0.67	7.37 *
	(0.76–2.94)	(1.30–27.81)	(0.50–1.43)	(0.58–3.60)	(0.41–1.78)	(0.15–2.43)	(0.34–1.38)	(0.31–4.07)	(0.79–2.79)	(0.49–6.28)	(0.36–1.25)	(0.91–59.50)
Rural health insurance * Urban living	0.84	0.16 **	0.81	0.43 *	0.86	1.41	1.16	0.58	0.56	0.36	1.08	0.13 *
	(0.38–1.86)	(0.03–0.79)	(0.44–1.50)	(0.17–1.13)	(0.36–2.06)	(0.32–6.27)	(0.52–2.60)	(0.15–2.22)	(0.27–1.19)	(0.10–1.37)	(0.52–2.27)	(0.02–1.04)
Observations	2199	2711	2191	2692	1280	1681	1256	1644	1274	1681	1281	1686

Notes: All models include the same variables as Table 3. BP: denotes blood pressure. Significance level: ** *p* < 0.05, * *p* < 0.10.
